# A methodology to determine the maximum value of weighted Gini–Simpson index

**DOI:** 10.1186/s40064-016-2754-8

**Published:** 2016-07-21

**Authors:** José Pinto Casquilho

**Affiliations:** Universidade Nacional Timor Lorosa’e, Rua Formosa, Díli, Timor-Leste; Centro de Ecologia Aplicada ‘‘Prof Baeta Neves’’, InBio, Instituto Superior de Agronomia, Universidade de Lisboa, Tapada da Ajuda, 1349-017 Lisbon, Portugal

**Keywords:** Weighted Gini–Simpson index, Critical solution, Feasibility of solution, Sequential procedures, Maximum point, Maximum value, Harmonic mean

## Abstract

Weighted Gini–Simpson index is an analytical tool that promises to be widely used concerning biological and economics applications, relative to the assessment of diversity measured by compositional proportions of a system defined with a finite number of elementary states characterized by positive weights. In this paper, a current literature review on the theme is presented and the mathematical properties of the index are outlined, focusing on the location of the maximizer (maximum point) and evaluation of the maximum value, with emphasis in the role of the Lagrange multiplier critical value—closely related with the harmonic mean of the weights—which is shown to be a barrier concerning the feasibility of the solution. Sequential procedures are presented, either backward or forward, which are used to obtain the correct values of the maximum point coordinates, thus allowing for the computation of the right maximum value of the index. Also, new theoretical results are provided, such as the calculus of limits and partial derivatives related to the critical solution, used to assess of the effectiveness of the algorithms herein proposed and discussed.

## Background

Weighted Gini–Simpson index may be considered a recent analytical tool since explicit mentions don’t seem available before the end of last century, and few empirical applications are yet available, though its number seems to be increasing fast. The problem that will be addressed in this paper concerns the location of maximum point and evaluation of the maximum value of the index, which are not trivial issues, as standard formulas referred to in the main literature just are straightforwardly applicable when a full set of inequalities are simultaneously verified. Otherwise, one has to proceed using algorithms as will be outlined in this paper. First, as background, the literature and main characteristics of Simpson and Gini–Simpson indices and of the correspondent weighted versions will be reviewed, followed by results and discussion of the methodology here proposed. Last, some conclusions are drawn.

### Simpson and Gini–Simpson indices

Considering a simplex of dimension *m* − 1 defined as $\Delta^{m - 1} = \left\{ {p_{i} \ge 0, i = 1, \ldots m;\sum\nolimits_{i = 1}^{m} {p_{i} } = 1} \right\}$, where the numbers *p*_*i*_ denote relative extension measures, usually probabilities or proportions,^1^ Simpson’s index, originally mentioned as a measure of the concentration of a classification (Simpson 1949) is evaluated with the formula $C = \sum\nolimits_{i = 1}^{m} {p_{i}^{2} }$ and its symmetric form *D* = 1 − *C* is usually named^2^ Gini–Simpson index (e.g., Rao 1982), and used as a measure of biological or phylogenetic diversity until today (e.g., Tryjanowski et al. 2015; Zaller et al. 2015; Brocchieri 2015), since we can rewrite the correspondent formula as $D = \sum\nolimits_{i = 1}^{m} {p_{i} \left( {1 - p_{i} } \right)}$ and interpret it associated to the probability that any two random individuals in a population are assigned to different populations or genetic clusters (e.g., Chybicki et al. 2014). In biological studies, more than seven decades ago, the term *p*_*i*_(1 − *p*_*i*_) was already mentioned as the contribution to the sampling variance due to any one species being sometimes observed and sometimes not (Fisher et al. 1943), later stated as the probability of interspecific encounters (Hurlbert 1971), or the probability of drawing two individuals of different type from a given collection (Gregorius and Gillet 2008). Good (1953), in a paper inspired by Alan Turing, defined parametric measures of heterogeneity of populations of *s* species, evaluated as $c_{m,n} = \sum\nolimits_{i = 1}^{s} {p_{i}^{m} \left( { - \log p_{i} } \right)^{n} }$ with *m*, *n* = 0,1,2,…. Using this formalism it follows that the case *c*_2,0_ is Simpson’s concentration index *C*, while *c*_1,1_ is Shannon (1948) statistical entropy.

The attribution to Corrado Gini of the earliest formulation of index *D*, more than a century ago, was related with the themes of variability devoted to the measurement of quantitative phenomena, and mutability, this one concerned with the measurement of qualitative phenomena. It is mentioned that Gini presented about 13 versions of the index (Ceriani and Verme 2012) and measuring variability is considered to be at the core of his procedure (de Finetti 1931). Sen (2005) says that Gini index opened the avenue for research in diversity analysis of qualitative categorical data models.

### Weighted Simpson and Gini–Simpson indices

It is not easy finding references relative to the use of weighted Simpson’s concentration index $C_{w} = \sum\nolimits_{i = 1}^{m} {w_{i} p_{i}^{2} }$ which seems to have been first explicitly stated and used as an inverse measure for antigenic diversity of a virus population (Nowak 1994); it was also used recently as a price-weighted biodiversity index of catch in freshwater fisheries in Malawi (Kasulo and Perrings 2006).

Sharma et al. (1978) discussed a non-additive information measure they named “generalized useful information of degree α” relative to a utility information scheme with *m* positive real numbers *w*_*i*_ also defined in the simplex Δ^*m*−1^, denoted parametrically^3^ as $I^{\alpha } \left( W \right) = \sum\nolimits_{i = 1}^{m} {w_{i} p_{i} \left( {p_{i}^{\alpha - 1} - 1} \right)/\left( {2^{1 - \alpha } - 1} \right)}$ with *α* ≠ 1, from which we can retrieve weighted Gini–Simpson index evaluating the semi-value of *I*^*α*^(*W*) with *α* = 2, obtaining $\sum\nolimits_{i = 1}^{m} {w_{i} p_{i} \left( {1 - p_{i} } \right)}$. The weights {*w*_*i*_}_*i*=1,…,*m*_ allow for taking into account different features related to ecological or economic values of species or other components of a system characterized by the proportions {*p*_*i*_}_*i*=1,…,*m*_ including the sampling effort, the phylogenetic distances or conservation values, to name a few possible applications.

Weighted Gini–Simpson (WGS) index seems to have been formerly conceived and studied as an analytical tool addressing the diagnosis of landscape mosaics composition (Casquilho 1999) where the maximum point of the index and its maximum value were discussed using Lagrange multipliers method. It was also stated as an approach used to assess inequality measures under the scope of utility theory (Sen 1999). Guiasu and Guiasu (2003) outlined conditional and weighted measures of ecological diversity presenting the formulas for the maximum value of WGS index and the optimal proportions, results which were further retrieved and generalized for triads of species (Guiasu and Guiasu 2010). Also, Casquilho (2009) discussed an issue relative to habitats valuation with complex numbers, conceiving weighted Gini–Simpson index as a sum of variances of interdependent Bernoulli variables indexed by positive characteristic values, either ecological or economic.

Several theoretical developments were presented in the following, from which stand out, concerning ecological or related biological fields: the application of weighted Gini–Simpson to assess ecomosaics compositional scenarios (Casquilho 2011); the application to biodiversity partitioning and measuring of diversity with respect to the pairs of species (Guiasu and Guiasu 2012); Ricotta et al. (2012), discussing Rao’s quadratic index under the scope of functional rarefaction, claim that their method is suitable to be extended to any concave diversity measure including WGS index; also, weighted Gini–Simpson index was said to be closely related to a unified framework based on Hill numbers concerning specific, phylogenetic, functional and other diversity measures (Chiu and Chao 2012; Chao et al. 2014); Guiasu and Guiasu (2014) proceeded with developments concerning the use of the index as a biodiversity assessment tool for interdependent species; Pavoine and Izsák (2014) formulated a new parametric index of diversity related to Rao’s quadratic entropy and discuss connections relative to other indices including WGS index; last, WGS index was combined with expected utility generating a non-expected utility device (Casquilho 2015). Other empirical studies or applications using WGS index will be mentioned in the discussion of results.

### Stating the problem

The problem addressed and discussed in this paper is that, in general, the maximum point coordinates of WGS index must be computed with a sequential procedure, because the formulas available in the most relevant literature concerning the issue (e.g., Guiasu and Guiasu 2003, 2010) are valid only within limited ranges of values of the set of predefined nonnegative weights {*w*_*i*_}_*i*=1,…,*m*_. If this remark is not scrutinized, the blind use of those formulas may inflate the proportions of the heaviest weighted components and lead to an erroneous “maximum value” evaluation, which can have pernicious consequences in subsequent normalization procedures or other inferences on the subject. Though the problem was previously mentioned (Casquilho 1999, 2009, 2011), it was not fully systematized and analytically focused, and one of the procedures proposed in this paper is new.

In fact, the problem at stake has an old root, as Jaynes (1957) had already pointed out that the negative term $- \sum p_{i}^{2}$ has the difficulty arising from the fact that conditional maxima cannot be found by a stationary property involving Lagrange multipliers, because the results do not, in general, satisfy the axiomatic condition *p*_*i*_ ≥ 0. We will see that it is such a kind of problem which is at the core of the subject that will be discussed in the following.

Next, the main mathematical properties of weighted Gini–Simpson index will be reviewed, focusing on the critical solution and the meaning of the Lagrange multiplier value as a parameter controlling the feasibility of the solution. Then, sequential backward and forward procedures or algorithms are outlined, associated with simple numerical examples illustrating the performance of the method, and last, results will be discussed.

## Review of the theoretical framework

Consider *m* interdependent Bernoulli variables *B*_*i*_ where *v*_*i*_ is a characteristic positive value, with associated probabilities *P*[*B*_*i*_ = *v*_*i*_] = *p*_*i*_ satisfying normalized measure space definition Δ^*m*−1^, and *P*[*B*_*i*_ = 0] = *q*_*i*_, with *q*_*i*_ = 1 − *p*_*i*_; thus $\sum\nolimits_{i = 1}^{m} {q_{i} = m - 1}$ equates the dimension of the simplex with *m* vertices. Computing the variance of *B*_*i*_ we obtain $Var\left( {B_{i} } \right) = v_{i}^{2} p_{i} \left( {1 - p_{i} } \right)$ from what follows the sum of variances $\sum\nolimits_{i = 1}^{m} {Var\left( {B_{i} } \right)} = \sum\nolimits_{i = 1}^{m} {v_{i}^{2} p_{i} \left( {1 - p_{i} } \right)}$. Renaming $v_{i}^{2}$ as $v_{i}^{2} = w_{i}$, the weighted Gini–Simpson index, measuring the variability of a system with such a characterization, is defined with the formula (1):

1 $$D_{w} = \mathop \sum \limits_{i = 1}^{m} w_{i} p_{i} \left( {1 - p_{i} } \right)$$

Index *D*_*w*_ is a continuous real function with domain in a compact set, the *m* − 1 simplex, what entails Bolzano–Weierstrass theorem to ensure that the index attains maximum and minimum values, as well as all the intermediate in its range. The inequality *D*_*w*_ ≥ 0 is easily seen to be true as the index is conceived as a sum of nonnegative terms, thus one can conclude straightforwardly that the minimum value *D*_*w*_ = 0 is reached at every vertex of the simplex (*p*_*j*_ = 1 and *p*_*i*_ = 0 if *i* ≠ *j*). Moreover, it is shown that index *D*_*w*_ is a concave function (e.g. Casquilho 1999; Guiasu and Guiasu 2010).

### Lagrange multiplier method and feasibility of solution

Also, index *D*_*w*_ is a real differentiable function, hence one can use the auxiliary Lagrangian function denoted as $L = \sum\nolimits_{i = 1}^{m} {w_{i} p_{i} \left( {1 - p_{i} } \right)} - \alpha \left( {\sum\nolimits_{i = 1}^{m} {p_{i} - 1} } \right)$ as an analytical tool for finding candidates to constrained extreme points of *D*_*w*_ (e.g. Bertsekas 1996) located in the hyperplane defined by the equation $\sum\nolimits_{i = 1}^{m} {p_{i} } = 1$. The calculus of generic partial derivative(s) in the variable(s) *p*_*i*_ entails the following result: $\partial L/\partial p_{i} = w_{i} - 2w_{i} p_{i} - \alpha$.

Searching the critical or stationary point of function *L* implies the system of equations:

2 $$p_{i} = \left( {w_{i} - \alpha } \right)/\left( {2w_{i} } \right)\quad {\text{for}}\; i = 1, \ldots ,m.$$

As the weights are positive real numbers by hypothesis (*w*_*i*_ > 0) one can state the immediate conclusion that the optimal proportions should verify the conditions $p_{i} \ge 0 \Leftrightarrow w_{i} \ge \alpha$, from what follows that the value of the Lagrange multiplier is a barrier, or limit value, concerning the feasibility of the solution evaluated with this method.

Computing the associated closure condition $\sum\nolimits_{i = 1}^{m} {p_{i} } = 1$ using (2), one obtains the critical value of the Lagrange multiplier:

3 $$\alpha^{ *} = \left( {m - 2} \right)\left/\left( {\mathop \sum \limits_{i = 1}^{m} \frac{1}{{w_{i} }}} \right)\right..$$

From what follows that the critical point evaluated combining (2) and (3) is defined by the equations:

4 $$p_{i}^{ *} = \frac{1}{2} + \left( {1 - \frac{m}{2}} \right)\left/\left( {w_{i} \mathop \sum \limits_{j = 1}^{m} \frac{1}{{w_{j} }}} \right)\quad {\text{for}}\; i = 1, \ldots ,m\right.$$

Formula (5) presented next, relative to the presumed maximum value of the index^4^ is the result of the evaluation of (1) replacing {*p*_*i*_}_*i*=1,…,*m*_ with the critical proportions defined in (4).

5 $$D_{w}^{ *} = \frac{1}{4}\mathop \sum \limits_{j = 1}^{m} w_{j} - \left( {1 - \frac{m}{2}} \right)^{2} \left/\left( {\mathop \sum \limits_{j = 1}^{m} \frac{1}{{w_{j} }}} \right)\right..$$

## Results and discussion

From Eq. (3) one can conclude immediately that for *m* ≥ 3 the inequality α* > 0 is verified and for *m* = 2 reduces to α* = 0 which implies the trivial result when the simplex is 1-dimensional: $p_{1}^{*} = p_{2}^{*} = 0.5$; also, the critical coordinates $p_{i}^{*}$ evaluated with (2) verify intrinsically the condition $p_{i}^{*} \le 1$, as the following equivalences show $p_{i}^{*} \le 1 \Leftrightarrow w_{i} - \alpha^{*} \le 2w_{i} \Leftrightarrow - \alpha^{*} \le w_{i}$ which is true because α* ≥ 0, the equality sign just holding for the 1-simplex; last, whether *w*_*i*_ = α* one gets the value $p_{i}^{*} = 0$. Next, it will be proved that the critical solution defined by Eq. (4) may not be feasible and, subsequently, cannot be used straightforwardly to evaluate the maximum value of the index as defined by formula (5).

### Analytical study of the critical point

Both formulas (4) and (5) are the right results whenever we have *w*_*i*_ ≥ α* for *i* = 1,…,*m* meaning that *m* inequalities must be verified simultaneously. Whether there is at least one value that verifies *w*_*i*_ < α* the evaluation of the optimal solution needs a revision in a sequential procedure, though that can only happen if *m* ≥ 4. In fact, successive replacements and simplifications allow for obtaining the equivalent results:

6 $$w_{i} > \alpha^{*} \mathop \Leftrightarrow \limits^{i = 1, \ldots ,m} w_{i} > \left( {m - 3} \right)\left/\left( {\mathop \sum \limits_{j \ne i} \frac{1}{{w_{j} }}} \right)\right.$$

Thus, for *m* = 3 one can see that the condition *w*_*i*_ > 0 is trivially verified and the critical proportions are properly defined as the maximizer coordinates:

7 $$p_{i}^{ *} = \frac{1}{2} - 1\left/\left( {2w_{i} \mathop \sum \limits_{j = 1}^{3} \frac{1}{{w_{j} }}} \right)\quad {\text{for}}\; {\text{i = 1,2,3}} \right..$$

A direct inspection of formula (4)—for which may be helpful to rewrite the denominator as $w_{i} \sum\nolimits_{j = 1}^{m} {\frac{1}{{w_{j} }}} = 1 + \sum\nolimits_{j \ne i} {\frac{{w_{i} }}{{w_{j} }}}$—shows that the critical proportion $p_{i}^{*}$ increases with the value of the corresponding *w*_*i*_ when all the other weights remain fixed, and, on the contrary, decreases with the increasing value(s) of other *w*_*j*_(*j* ≠ *i*).

The calculus of limits on formulas (4) for *m* ≥ 3 also clarifies the issue: $\mathop {\lim }\nolimits_{{w_{i} \to + \infty }} p_{i}^{ *} = \mathop {\lim }\nolimits_{{w_{i} \to + \infty }} \left( {\frac{1}{2} + \left( {1 - \frac{m}{2}} \right)/\left( {1 + \mathop \sum \nolimits_{j \ne i} \frac{{w_{i} }}{{w_{j} }}} \right)} \right) = \frac{1}{2}$, and from this result one can conclude that there is a supremum (least upper bound) for the optimal point coordinates: $p_{i}^{*} < 0.5$ in the context (and $p_{i}^{*} = 0.5$ if *m* = 2); also, $\mathop {\lim }\nolimits_{{w_{i} \to 0^{ + } }} p_{i}^{ *} = 3/2 - m/2$ which is the same result that is obtained when *w*_*i*_ is fixed and all the other weights *w*_*k*_(*k* ≠ *i*) tend simultaneously to positive infinite. For example, if *m* = 5 we get the result $\mathop {\lim }\nolimits_{{\left\{ {w_{k} } \right\}_{k \ne i} \to + \infty }} p_{i}^{*} = - 1$. This negative value shows that when applying formulas (4) we can obtain non-feasible solutions, becoming negative without bound as the dimension of the simplex increases.

Also, the calculus of partial derivatives in Eq. (4) shows that the value $p_{i}^{ *}$ increases with the corresponding weight *w*_*i*_ and decreases when any other weight *w*_*k*_ increases. In fact, one can see that, for *m* ≥ 3, the following inequalities hold:

$$\frac{{\partial p_{i}^{ *} }}{{\partial w_{i} }} = \left( {\frac{m}{2} - 1} \right)\left( {\mathop \sum \limits_{j \ne i} \frac{1}{{w_{j} }}} \right)\left( {w_{i} \mathop \sum \limits_{j = 1}^{m} \frac{1}{{w_{j} }}} \right)^{ - 2} > 0\quad {\text{and}}\quad \frac{{\partial p_{i}^{ *} }}{{\partial w_{k} }} = \left( {1 - \frac{m}{2}} \right)\left( {w_{i} \mathop \sum \limits_{j = 1}^{m} \frac{1}{{w_{j} }}} \right)^{ - 2} \left( {\frac{{w_{i} }}{{w_{k}^{2} }}} \right) < 0.$$

So, what happens if there is any *w*_*i*_ < α*? Then, the corresponding critical value $p_{i}^{*}$, although lying in the hyperplane defined by the equation $\sum\nolimits_{i = 1}^{m} {p_{i}^{*} } = 1$ is not located in the simplex, and the solution is not feasible. In the general case with *m* ≥ 4 there will be *m*′ ≥ 3 non-null optimal proportions as stated by Eq. (7) and *m* − *m*′ null coordinates. In some well balanced sets of weights it may happen that *m*′ = *m* but it is a particular case, not the general one.

### Sequential forward procedure

Next, it is outlined a sequential forward procedure to reach the maximum point (maximizer) of the index. First, one sorts the weights in a decreasing order: $w_{\left( 1 \right)} \ge w_{\left( 2 \right)} \ge \cdots \ge w_{\left( m \right)}$. From Eq. (7), combined with the evaluation of limits and partial derivatives, it is known that it is guaranteed that the three highest weighted components will be in the optimal solution, with strictly positive proportions; the fourth highest weighted component is the first candidate to have a null value; then, one computes $\alpha_{4}^{*} = 2/\left( {\sum\nolimits_{i = 1}^{4} {1/w_{\left( i \right)} } } \right)$ and if $w_{\left( 4 \right)} \le \alpha_{4}^{*}$ stop; hence $p_{\left( 4 \right)}^{*} = \cdots = p_{\left( m \right)}^{*} = 0$ and the number of vertices is set *m*′ = 3; otherwise one has $w_{\left( 4 \right)} > \alpha_{4}^{*}$ and proceeds computing $\alpha_{5}^{*} = 3/\left( {\sum\nolimits_{i = 1}^{5} {1/w_{\left( i \right)} } } \right)$; whether $w_{\left( 5 \right)} \le \alpha_{5}^{*}$ stop, and reset the values $p_{\left( 5 \right)}^{*} = \cdots = p_{\left( m \right)}^{*} = 0$ with *m*′ = 4; otherwise, $w_{\left( 5 \right)} > \alpha_{5}^{*}$ and one proceeds until obtaining $w_{\left( k \right)} \le \alpha_{k}^{*}$, then stop, setting $p_{\left( k \right)}^{*} = \cdots = p_{\left( m \right)}^{*} = 0$; hence *m*′ = *k* − 1; in any case, the maximizer is located in a *m*′-face of the original *m* − 1 simplex.

Now, formulas (4) and (5) may be used replacing *m* by *m*′ and calculating the optimal proportions and the maximum value of the index *D*_*w*_ with the corresponding set of weights—all the remaining optimal proportions being null and the respective weights discarded from the evaluation.

Exemplifying with a relatively small dimension *m* = 5, which enables the lower bound of a pseudo-optimal coordinate to be −1, as was shown in the calculus of the limits in the previous section. If the values of the weights are *w*_(1)_ = 5, *w*_(2)_ = 4, *w*_(3)_ = 3, *w*_(4)_ = 2 and *w*_(5)_ = 1, then the value of the Lagrange multiplier computed with (3) and all weights (*m* = 5) gives the result α* = 1.3139 implying that *w*_(5)_ < α*; using the sequential procedure, one calculates $\alpha_{ 4 }^{*} = 1. 5585$ and as $w_{\left( 4 \right)} > \alpha_{4}^{*}$ hence $p_{\left( 5 \right)}^{*} = 0$ and formulas (4) and (5) may be applied with *m*′ = 4, discarding *w*_(5)_ = 1 from the calculations, giving the results of the optimal proportions: $p_{\left( 1 \right)}^{*} = 0.3441$, $p_{\left( 2 \right)}^{*} = 0.3052$, $p_{\left( 3 \right)}^{*} = 0.2403$, $p_{\left( 4 \right)}^{*} = 0.1104$ and $p_{\left( 5 \right)}^{*} = 0$. The maximum of the index in this case evaluates to $D_{w}^{*} = 2.7208$.

Changing the weights to be: *w*_(1)_ = 50, *w*_(2)_ = 40, *w*_(3)_ = 30, *w*_(4)_ = 2 and *w*_(5)_ = 1, and using the sequential forward procedure one computes $\alpha_{ 4 }^{*} = 3.4582$ and verify that $w_{\left( 4 \right)} < \alpha_{4}^{*}$ hence sets $p_{\left( 4 \right)}^{*} = p_{\left( 5 \right)}^{*} = 0$ and *m*′ = 3, thus discarding *w*_(4)_ and *w*_(5)_, proceeding to evaluate the non-null coordinates with Eq. (7), so obtaining the results: $p_{\left( 1 \right)}^{*} = 0.3724$, $p_{\left( 2 \right)}^{*} = 0.3404$ and $p_{\left( 3 \right)}^{*} = 0.2872$. In this case, the maximum value is $D_{w}^{*} = 26.808$ and, in this example, whether formula (5) was used blindly with all the original weights (*m* = 5) one would obtain the wrong pseudo-maximum value of 29.324 is misvalued about 10 % relative to the true value. When the dimension of the simplex increases, and the weights are disparate, this type of error could get worse in a kind of curse of dimensionality.

### Sequential backward procedure

Whether the forward procedure previously discussed helps checking the consistency of the problem stated by the feasibility condition expressed in inequalities (6), one can see that a sequential backward procedure is more effective, applying directly Eq. (4) and nothing else. Adopting the same ordering $w_{\left( 1 \right)} \ge w_{\left( 2 \right)} \ge \cdots \ge w_{{\left( {m - 1} \right)}} \ge w_{\left( m \right)}$ begin computing $p_{\left( m \right)}^{*}$ and if $p_{\left( m \right)}^{*} > 0$ then Eq. (4) are proper, all the coordinates can be calculated directly and also Eq. (5) applies straightforwardly with no problem; otherwise, if $p_{\left( m \right)}^{*} < 0$ then set $p_{\left( m \right)}^{*} = 0$ withdraw *w*_(*m*)_ from further calculations and compute $p_{{\left( {m - 1} \right)}}^{*}$ with Eq. (4) modified with *m*′ = *m* − 1 and the corresponding set of weights; proceed with the same reasoning, recurring, until one finds an order (*k*) such that $p_{\left( k \right)}^{*} > 0$, then stop; set all null coordinates $p_{{\left( {k + 1} \right)}}^{*} = \cdots = p_{\left( m \right)}^{*} = 0$, and Eq. (4) apply with dimension reset as *m* = *k* evaluated with the corresponding weights {*w*_(*i*)_}_*i*=1,…,*k*_.

Retrieving the numerical examples from the previous section one has again *w*_(1)_ = 5, *w*_(2)_ = 4, *w*_(3)_ = 3, *w*_(4)_ = 2 and *w*_(5)_ = 1 then computing $p_{\left( 5 \right)}^{*}$ with formula (4) and *m* = 5 one gets $p_{\left( 5 \right)}^{*} = - 0.15693 < 0$; so, one sets $p_{\left( 5 \right)}^{*} = 0$, discards *w*_(5)_ = 1 and evaluates $p_{\left( 4 \right)}^{*}$ with *m*′ = 4 and {*w*_(*i*)_}_*i*=1,…,4_; next result is $p_{\left( 4 \right)}^{*} = 0.11039 \cong 0.1104 > 0$, so stop; all the other coordinates can be calculated now with Eq. (4) and *m*′ = 4.

With the other example relative to the set of weights *w*_(1)_ = 50, *w*_(2)_ = 40, *w*_(3)_ = 30, *w*_(4)_ = 2 and *w*_(5)_ = 1 one has the following sequence: in the first step computes $p_{\left( 5 \right)}^{*} = - 0.45037 < 0$ so one discards *w*_(5)_ = 1, sets *m*′ = 4 and proceeds evaluating $p_{\left( 4 \right)}^{*}$ with the corresponding weights $\left\{ {w_{\left( i \right)} } \right\}_{i = 1, \cdots ,4}$ obtaining $p_{\left( 4 \right)}^{*} = - 0.36455 < 0$; then, one sets $p_{\left( 4 \right)}^{*} = 0$ with *m*′ = 3 and proceeds evaluating $p_{\left( 3 \right)}^{*} , p_{\left( 2 \right)}^{*}$ and $p_{\left( 1 \right)}^{*}$ with the set {*w*_(*i*)_}_*i*=1,…,3_ and Eq. (7), obtaining, for example, the value $p_{\left( 3 \right)}^{*} = 0.28723 \cong 0.2872$; and the maximum value of *D*_*w*_ now can be evaluated using Eq. (5) with *m*′ = 3.

## Discussion

The index *D*_*w*_ is a continuous real function defined in a compact domain and its range is $0 \le D_{w} \le D_{w}^{*}$, the minimum value *D*_*w*_ = 0 occurring in every vertex of the simplex Δ^*m*−1^. The maximum value of the index denoted $D_{w}^{*}$ has to be evaluated verifying the feasibility condition expressed by inequalities (6) before applying straightforwardly Eq. (5). In general, except for very specific and balanced sets of weights, the maximum point of *D*_*w*_ will not occur in the interior of the simplex but in a *k*-face with 3 ≤ *k* < *m*, as was shown by the theoretical results followed by sequential procedures and illustrated with numeric examples, leading to some null optimal coordinates.^5^ Obviously, the maximum value of WGS index could also be computed as $D_{w}^{*} = \mathop \sum \nolimits_{i = 1}^{k} w_{i} p_{i}^{*} \left( {1 - p_{i}^{*} } \right)$ thus avoiding Eq. (5), but that still implies checking the feasibility condition as the summing procedure just applies for positive proportions.

The optimal proportions $p_{i}^{*}$ are insensitive to a change in unities: the positive linear transformation *u*_*i*_ = *cw*_*i*_, *c* > 0 implies that the critical solution remains the same and the new value of the Lagrange multiplier is also linearly transformed to be α** = cα* entailing that the feasibility condition (6) remains unchanged. The optimal value of the Lagrange multiplier α* defined in (3) is closed related to the harmonic mean of the weights. How can we justify that α* has numerator *m* − 2 instead of *m*? It seems that the most appealing interpretation is that when discussing result (3) we deduced that for *m* = 2 the value α* vanishes and the maximum point is fixed: $\left( {p_{1}^{*} ,p_{2}^{*} } \right) = \left( {0.5,0.5} \right)$, independent of the weights. So, *m* − 2 is the number of relevant weights that affect the subsequent calculation of the maximum point coordinates and maximum value of the index.

The problem addressed in this paper is particularly important when the evaluation of $D_{w}^{*}$ aims to be used in further normalization assessments with range $0 \le D_{w} /D_{w}^{*} \le 1$ and an erroneous computation of the maximum value can induce wrong conclusions when comparing different compositional systems. There are several empirical studies that use the maximum value of WGS index as a reference for further normalization assessments: besides Guiasu and Guiasu (2010) numeric examples such as the one relative to 10 species in two habitats with data retrieved from Jost et al. (2010), also Subburayalu and Sydnor (2012) used formula (5) when assessing street tree diversity in four Ohio communities and, probably, the feasibility condition here discussed was not checked. Weighted Gini–Simpson goes on being mentioned (e.g., Niane et al. 2014) and the problem handled in the present article seems to become relevant for the next future.

## Conclusions

In this paper it was summarized an issue that seems to be relevant in the field of diversity measures: the proper evaluation of the maximum point and maximum value of weighted Gini–Simpson index. The main literature on the subject does not refer to the feasibility condition here discussed, what can involve consequent wrong results in applications. New theoretical results concerning the analytical study of the critical solution are provided, such as the calculus of limits and partial derivatives, as well as are sketched forward and backward procedures conceived to solve the issue at stake, also illustrated with numeric examples.

## Footnotes

1 In biodiversity studies, proportions of populations in a community are commonly designated as relative abundances of species.

2 Also referred to as Simpson’s index of diversity (e.g., Crist et al 2003; Niane et al. 2014).

3 Aggarwal and Picard (1978) say that Emptoz had outlined an equivalent formula in 1976.

4 Here we use the notation of Guiasu and Guiasu (2003); an equivalent formula with a different notation may be found in Casquilho (1999:121,122).

5 As it can happen with other biodiversity indices (e.g., Pavoine and Izsák 2014).
